# COVID-19 mRNA Based Vaccine Immune-Response Assessment in Nursing Home Residents for Public Health Decision

**DOI:** 10.3390/vaccines9121429

**Published:** 2021-12-02

**Authors:** David San Segundo, Alejandra Comins-Boo, Patricia Lamadrid-Perojo, Juan Irure-Ventura, José María Castillo-Otí, Reinhard Wallman, Jorge Calvo-Montes, José Manuel Méndez-Legaza, Carmela Baamonde-Calzada, Isabel Sánchez-Molina, Marina Lecue-Martínez, Silvia Ventisca-Pérez, Ana Batlle-López, Marcos López Hoyos

**Affiliations:** 1 Immunology Department, University Hospital Marqués de Valdecilla, 39008 Santander, Spain; david.sansegundo@scsalud.es (D.S.S.); alejandra.comins@scsalud.es (A.C.-B.); juan.irure@scsalud.es (J.I.-V.); 2 Transplantation and Autoimmunity Laboratory, Research Institute “Marqués de Valdecilla” (IDIVAL), 39008 Santander, Spain; plamadrid@idival.org; 3 Epidemiological Monitoring and Intervention Unit of Liencres Hospital, 39120 Piélagos, Spain; josemaria.castillo@scsalud.es (J.M.C.-O.); marina.lecue@scsalud.es (M.L.-M.); silvia.ventisca@scsalud.es (S.V.-P.); 4 Comunitary Health Group Research Institute “Marqués de Valdecilla” (IDIVAL), 39008 Santander, Spain; 5 Nursing Department, School of Nursing, University of Cantabria, 39008 Santander, Spain; 6 Department of Public Health, Cantabrian Government, 39008 Cantabria, Spain; reinhard.wallmann@scsalud.es; 7 Microbiology Department, University Hospital Marqués de Valdecilla, 39008 Santander, Spain; jorge.calvo@scsalud.es (J.C.-M.); josemanuel.mendez@scsalud.es (J.M.M.-L.); 8 Epidemiology and Molecular and Pathogenic Mechanisms of Infectious Diseases and Clinical Microbiology Group Research Institute “Marqués de Valdecilla” (IDIVAL), 39008 Santander, Spain; 9 Clinical Analysis Department, Sierrallana Hospital, 39300 Torrelavega, Spain; carmela.baamonde@scsalud.es; 10 Clinical Analysis Department, Laredo Hospital, 39770 Laredo, Spain; isabel.sanchez-molina@scsalud.es; 11 Haematology Department, University Hospital Marqués de Valdecilla, 39008 Santander, Spain; mana.batlle@scsalud.es; 12 Haematologic Neoplasms and Haematopoietic Stem Cells Transplantation, IDIVAL, 39008 Santander, Spain; 13 Laboratory Informatic Management System Coordinator (LISCAN), Servicio Cantabro de Salud (SCS), Consejeria de Sanidad, 39008 Cantabria, Spain; 14 Molecular Biology Department, University of Cantabria, 39011 Santander, Spain

**Keywords:** mRNA vaccine, SARS-CoV-2, cellular-immune response, nursing home residents, public health

## Abstract

Nursing home residents (NHR) have been targeted as a vaccination priority due to their higher risk of worse outcome after COVID-19 infection. The mRNA-based vaccine BTN2b2 was first approved in Europe for NHRs. The assessment of the specific vaccine immune response (both humoral and cellular) at long term in NHRs has not been addressed yet. A representative sample of 624 NHR subjects in Northern region of Spain was studied to assess immune response against full vaccination with BTN2b2. The anti-S1 antibody levels and specific T cells were measured at two and six months after vaccination. 24.4% of NHR had a previous infection prior to vaccination. The remaining NHR were included in the full vaccination assessment group (FVA). After two months, a 94.9% of the FVA presented anti-S1 antibodies, whereas those seronegative without specific cellular response were 2.54%. At long-term, the frequency of NHR within the FVA group with anti-S1 antibodies at six months were 88.12% and the seronegative subjects without specific cellular response was 8.07%. The cellular immune assays complement the humoral test in the immune vaccine response assessment. Therefore, the cellular immune assessment in NHRs allows for the fine tuning of those seronegative subjects with potential competent immune responses against the vaccine.

## 1. Introduction

The Nursing home residences have been sensitive locations where the SARS-CoV-2 (COVID-19) virus has spread rapidly [1]. The subjects with worse prognosis and higher risk of mortality were those with associated comorbidities and the elderly, this latter group being a priority in the vaccination strategies [2]. However, the different vaccination rates within different groups as students [3] and NHRs [4] could compromise the vaccination success. The vaccination against SARS-CoV-2 with BNT162b2 is safe and effective, conferring a 95% protection against COVID-19 in subjects over 16 years old [5], and reducing hospitalization and mortality.

New SARS-CoV-2 variants have emerged and, despite vaccination, virus isolations in nursing home residences have been detected. Thus, despite vaccination, susceptible subjects should be identified early in order to proceed with new strategies of prevention and immunization. Strategies for public health control have been proposed, such as a third vaccine dose, despite evidence that the two dose strategy continues to be most effective against variants of concern. However, no deep immunological studies have been addressed in order to identify those fully vaccinated elderly subjects at risk of infection. The aim of this strategy is to identify low responders within patients with impaired immunities such as immunodeficiency, autoimmunity, cancer, transplantation or immunosenescence. The most precise way to measure immunological response to vaccination would include both cellular and humoral specific responses. Recently, the utility of both cellular and humoral immunity assessment to identify low responders within kidney [6], liver and heart transplant recipients [7], in addition to hematologic malignancies [8], autoimmunity [9] and the elderly [10] has been demonstrated.

The assessment of humoral immune response after the BNT162b2 vaccine in NHR has been recently published [11]. This group observed 30% of low post–vaccine anti S–RBD antibody levels, and importantly 3% of NHR remained seronegative after six weeks of the second shot. However, neither data about cellular immunity nor risk evaluation for symptomatic COVID-19 was performed. As far as we know, no studies of both humoral and cellular immune responses to the SARS-CoV-2 virus vaccine in NHRs have been previously addressed. 

In this study, to detect a possible break in the protection from vaccination, we performed early and medium–term assessments of specific BNT162b2 vaccine immune responses (including both humoral and cellular immunity) in NHR. Moreover, a risk assessment of positive PCR post-vaccination (PPPV) was evaluated. The detection of fully vaccinated NHRs without humoral and cellular response could further suggest the need to boost vaccine dose to enhance immune response.

## 2. Materials and Methods

### 2.1. Data Collection

**Figure 1:**
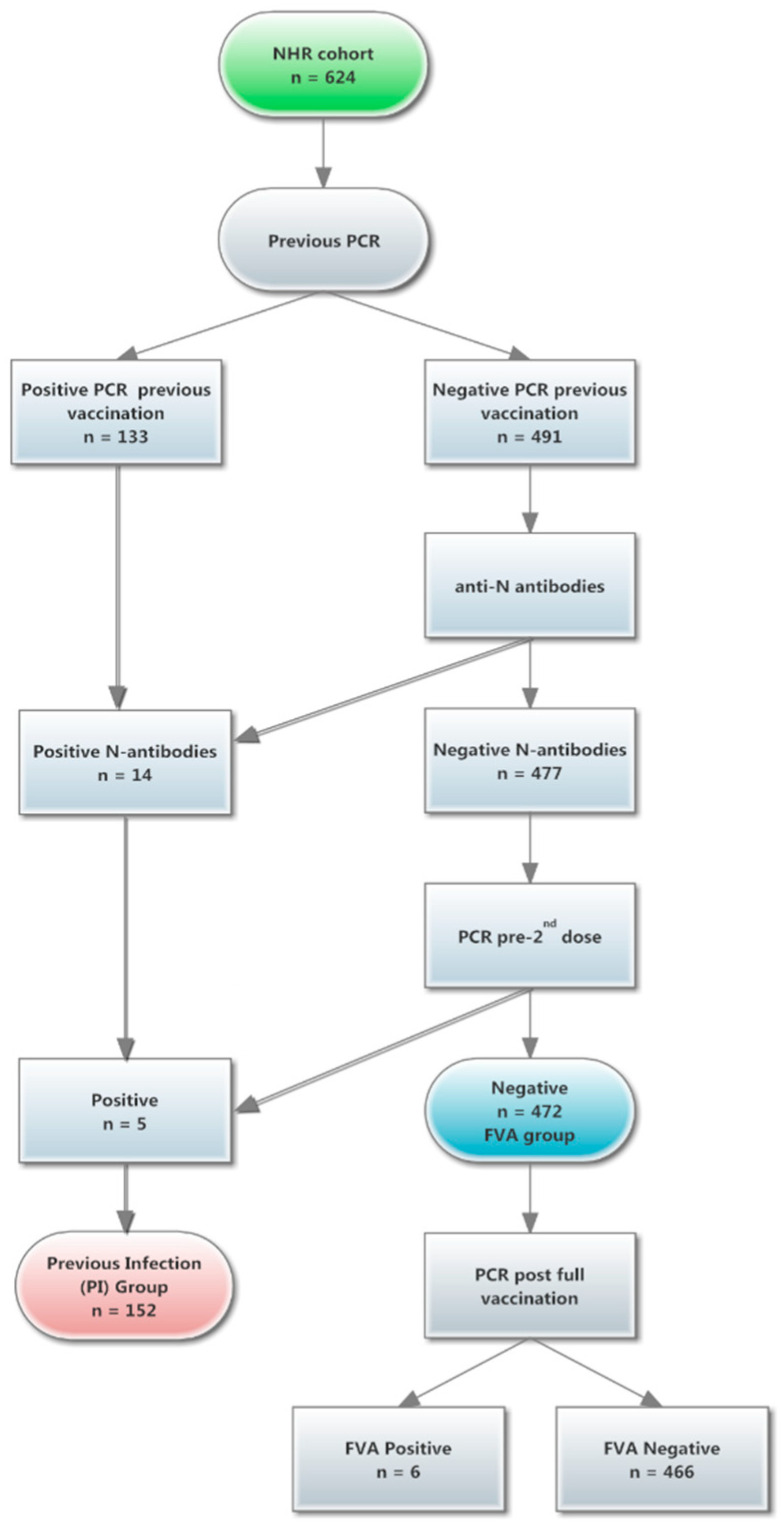
Study design. A total of 624 nursing home residents (NHR) were eligible for the study. Two different groups were defined: previous infection (PI) and full vaccination assessment (FVA).

Nursing home residents (NHR) in the Cantabrian region (North of Spain) were vaccinated following both the public health recommendations and European guidelines [12] from January 2021 to March 2021. The total NHRs at the moment of the study in the Cantabrian region were 6598 subjects. The sample size was calculated to estimate 85% of the population with a confidence interval of 95% and an accuracy of +/−3%. A total of 641 subjects were enrolled in the study, but 17 subjects were not feasible to get samples. Finally, the early assessment of immune vaccine response was conducted in a sample of 624 residents selected for the study, covering all residents in the region (57 nursing home residences), and all the subjects received two doses of the BNT162b2 vaccine. Further long-term assessments after six months of vaccination was performed in a subgroup of 558 NHR. The study design is summarized in Figure 1. The sample size was chosen by simple random sampling from among the total NHR population.

Sequential real time PCR was performed every two weeks after full vaccination to monitor potential infections. Moreover, in those patients with suspicion of infection or close contact a PCR or antigen test was performed; in the case of negative results, a further PCR test was undertaken.

Our Unified Laboratory Management System in Cantabria (LISCAN) integrated with a nursery home platform (GESCARE) and a unified citation system (TICARES) were instrumental to determine in real time the appropriate diagnostic path to be followed for each patient.

### 2.2. SARS-CoV-2 Anti-S Antibodies Detection

Serum samples were evaluated after two and six months of complete BNT162b2 for the detection of anti-SARS-CoV-2 S1 (RBD) protein-targeting (anti-S1) and specific anti-N IgG antibodies by chemiluminescent microparticle immunoassay (CLIA) using the SARS-CoV-2 IgG II Quant Assay on the Alinity I (Abbott, Chicago, IL, USA) following the manufacturer’s indications. Results were expressed as arbitrary units (AU) per mL (AU/mL). Values higher than 50 AU/mL for anti-S1 and higher than 1.5 AU/mL for anti-N were considered positive.

### 2.3. SARS-CoV-2 T-Specific Response Assessment by Flow Cytometry

The specific cellular immune response against vaccination was assessed as previously described [13]. Briefly, peripheral blood mononuclear cells (PBMCs) from sodium-heparinized blood were isolated by Ficoll gradient and cultured at 10^6^/mL in TexMACS medium (MiltenyiBiotec, BergischGladbach, Germany) during 24 h at 37 °C in a flat-bottom 96-well plate in 0.1% DMSO, PepTivator SARS-CoV-2 Prot S, Prot M and Prot N (1 ug/mL) and Dynabeads Human T activator CD3/CD28 (GibcoThermo Fisher Scientific Baltics UAB, Lithuania) as a positive control. After incubation, the PBMCs were washed and stained with the following monoclonal antibodies: anti-CD3 (FITC) clone UCHT 1 (Inmunotech SAS Beckman Coulter, Marseille, France), anti-CD4 (APC–Vio 770) clone VIT4 (MiltenyiBiotec, BergischGladbach, Germany), anti-CD8 (ECD) clone SFCI21Thy2D3 (Beckman Coulter, 737659, Brea, CA, USA), anti-CD134 (PE) clone 134–1 (Cytognos, Salamanca, Spain), and anti-CD25 (PE–Cy7) clone 2A3. The stained PBMCs samples were washed with phosphate buffer saline (PBS) 150 µL and centrifuged for five minutes at 1800 rpm. Finally, 2 µL of 7–Aminoactinomycin D (7–AAD) staining solution (Tonbo Biosciences, San Diego, CA, USA) and 90 µL of PBS were added before the samples were acquired on the CytoFLEX Flow Cytometer (Beckman Coulter). The results were expressed as the frequency in the activation induced molecules (CD25^+^CD134^+^) ratio obtained after specific activation to negative non-stimulated control. A ratio > 3 in one of the specific SARS-CoV-2 peptides was considered positive as previously described [13], and representative examples are summarized in Figure S1. 

### 2.4. Statistical Analysis

Statistical analysis was performed using SPSS version 19.0 (Chicago, IL, USA) and Graph Pad Prism (software 6.0 version). The distribution of continuous variables was assessed using the Kolmogorov-Smirnov/Shapiro-Wilk tests. Comparisons were based on U-Mann Whitney and the Student’s t test, as corresponded. Results were expressed as median (interquartile range, (IQR) or mean ± standard deviation, as appropriate. Receiver operation characteristic (ROC) analysis was performed to calculate the cutoff value of anti S-RBD levels after vaccination to better discriminate the risk of PPPV. A two-sided *p*-value < 0.05 was considered statistically significant.

## 3. Results

### 3.1. NHR Participants

The mean age of the NHR cohort was 80.56 years (standard deviation, SD, 13.51), of which 67.6% were female. All residents were fully vaccinated with the BNT162b2 vaccine between January and March 2021. The second dose was administered three weeks after the first dose, independently of previous COVID-19 infection. 

The NHR cohort was divided into two groups in order to assess the sensitization status (Figure 1). First was the previous COVID19 infection (PI) group, which included a total of 152 subjects, and 133 of them had positive PCRs before administration of the first vaccine dose. Five were PCR positive between the first and the second dose. In this group, we have also included 14 residents who presented detectable anti-N antibodies as a marker of prior infection.

Secondly, the remaining subjects (*n* = 472) without previous infection were included in the full vaccine assessment (FVA) group. From this group, a total of 17 residents had a positive PCR (FVA+), two early (median 89 days) and 15 late (at six months, median 209 days) after second dose. Twelve out of 17 (70.6%) subjects from the FVA+ group were asymptomatic, whereas five subjects with symptoms required hospitalization (1.06% of the FVA group), and one of them died without a clear association with COVID-19 disease (non-compatible radiologic signs).

### 3.2. Early Assessment of Quantitative Humoral Vaccine Response in NHR Cohort

**Figure 2:**
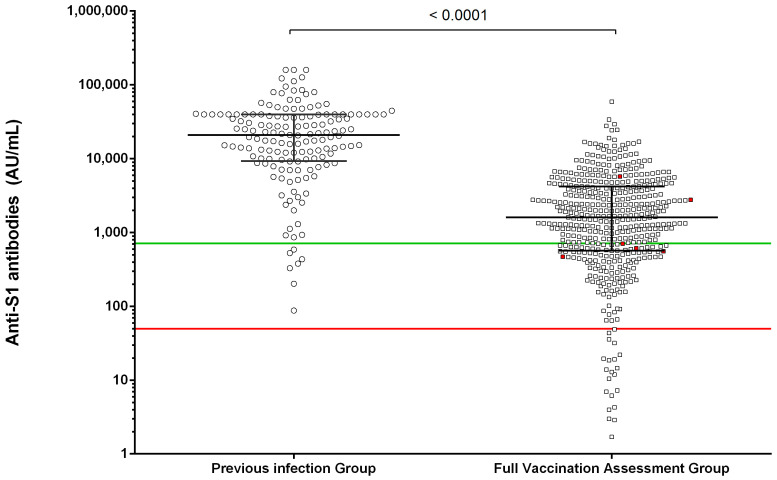
Comparison of anti-S1 antibody levels after two months of BNT162b2 full vaccination in nursing home residents (NHR). The anti-S1 antibody level in the previous infection group (white circles) and full vaccination assessment group (white squares) is depicted. The anti-S1 levels below the red line at 50 AU/mL indicates those NHR identified as seronegative after full vaccination. Whereas a value below 715 AU/mL of anti-S1 antibody level (green line) after two months of vaccination shows patients at risk of infection (red squares). The mean differences were assessed by Student’s *t*-test.

To assess the vaccine response in NHR, the level of anti-S1 antibodies was measured. A wide range of detection was observed (<50 to >40,000 AU/mL) in the NHR cohort. An increased level of anti-S1 antibodies in PI group compared with FVA group (median, (IQR): 19,669 (8534–40,000) vs 1611 (570.9–4219), respectively (*p* < 0.001) was observed after two months of vaccination (Figure 2).

To fully characterize the anti-S1 antibody level, different groups were defined based in anti-S1 levels as previously described [9] in seronegative: <50; low responders: 50–1050; moderate: 1050–4160; and high responders: >4160 AU/mL. The PI group demonstrated higher levels since the frequency of anti-S1 antibody levels in each group was = 0%; 6.25%; 4.86% and 88.89% respectively, whereas in the FVA group it was = 4.80%; 32.78%; 36.32% and 26.10%, respectively (Chi square test, *p* < 0.001). 

Subsequently, to assess the potential risk of infection post-vaccination, the analysis of anti-S1 antibody levels was addressed in the FVA group. The median (IQR) of anti-S1 antibody levels in the FVA+ group was decreased compared with the FVA- group (662 (536–3515) vs 1611 (570.9–4219), *p* = NS). However, after ROC analysis a value of 715 AU/mL identified those patients with risk of infection after vaccination (with a 70.5% and 66.7%, sensitivity and specificity, respectively), the hazard ratio of 1.023 (0.993–1.054), *p* = 0.069 (Figure 2).

### 3.3. Early Evaluation of Immunization Status in NHR

All NHR were fully vaccinated despite previous COVID-19 contact. The assessment of humoral response was performed two months after the second dose. A total of 600 NHR out of 624 had anti-S1 antibodies, reflecting a specific response against SARS-CoV-2 and/or vaccination in 96.2% of the NHR cohort. The remaining 24 patients (3.8%) had neither anti-S1 nor anti-N antibodies after full dose vaccination.

The level of anti-S1 antibodies was significantly higher in the PI group with respect to the FVA (Figure 2), and to eliminate the potential bias of a previous contact with the virus and to perform a strict vaccine assessment, only the FVA group was further investigated. 

Twenty-four subjects of the FVA group (24/472, 5.08%) did not show specific antibody response, neither anti-S1 antibodies nor anti-N antibodies after two months of full vaccination. In these seronegative residents, a specific CD4+ T cell immune test was performed to assess the vaccine response. Twelve out of twenty–four (50%) did not show any specific cellular immune response (complete non-responders) against the SARS-CoV-2 peptide pool (anti-S, anti-M or anti-N). The clinical parameters of these early non-responders are summarized in Table S1. Overall, the vaccine responders (anti-S1 antibodies and/or specific cellular immune test) within the FVA group were 97.42%.

To assess the potential utility of cellular specific immunity against SARS-CoV-2 in order to identify break of protection of vaccine in seronegative subjects, the risk of infection should be assessed within complete non-responders’ subjects. In our cohort, none of the non-responders within the FVA group had a positive PCR after vaccination (Figure 2).

### 3.4. Immunization Status in NHR at 6 Months after Full Vaccination

**Figure 3:**
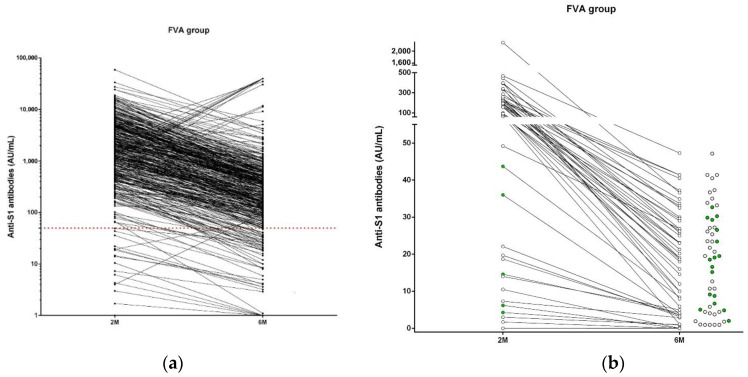
Comparison of anti-S1 antibody levels and cellular immunity after two and six months of BNT162b2 full vaccination in nursing home residents (NHR) included in Full Vaccination Assessment (FVA) group. The level of anti-S1 antibodies was compared in NHR at two and six months after full vaccination in the FVA group. The red line shows the anti-S1 levels at 50 AU/mL, subjects with anti-S1 values below this line were identified as seronegative (**a**). All seronegative NHR after six months of vaccination were assessed for cellular immunity; the green circles show positive specific cellular immunity whereas the white circles show those without a cellular-specific response to vaccination (**b**).

Finally, in order to investigate the degree of immunization in fully vaccinated NHR, a subgroup of the initial cohort (*N* = 558) was tested for anti-S1 antibodies. A total of 137/558 were of the PI group, the remaining 421 were in FVA group. Fifty out of 421 FVA (11.88%) NHR were seronegative at six months of vaccination, 16 of them were also seronegative at two months of vaccination. In 17 out of the remaining 34 NHR who become seronegative, a specific cellular immunity was maintained (50.0%) at six months post vaccination. In Figure 3a, the level of anti-S1 antibodies after two and six months of vaccination are shown. Those NHR seronegative in each timepoint were tested for specific cellular immunity to fully investigate specific immune response against the SARS-CoV-2. Full negative specific immune response to the coronavirus peptides was observed in 12/624 (1.92%) and 33/558 (5.91%) of the NHR cohort at two and six months, respectively (Figure 3b). Considering only those included in the FVA group, the frequencies of subjects without vaccination response were 12/472 (2.54%) and 34/421 (8.07%) at two and six months, respectively. The clinical parameters NHR seronegative at 6 months are summarized in Table S2.

## 4. Discussion

The NHR subjects have been especially vulnerable to COVID-19. Public health authorities have to design strategies to protect them [12]. With the approval of a vaccine, the NHR were the first group to get vaccinated. However, different strategies were carried out in different countries. In the UK a single dose was established to reach higher numbers of vaccination in less time. Recently, the humoral and cellular immune response to a single dose of BNT162b2 in residential care homes showed delayed antibody responses in those infection–naïve residents, suggesting that giving a second dose early would be required [14]. 

In our cohort, 22% of NHR had previous contact with the COVID-19 virus. Due to natural immunity, the boost after mRNA vaccination increased anti-S1 antibody levels as previously described [11]. Individuals with previous contact before vaccination had increased levels of anti-S1 antibodies after full dose BNT162b2 vaccination [15]. In our cohort of NHR we have corroborated these data, despite the difference in the mean age of the subjects (80 years in the present study vs 41.89 [15]. This is in line with the generation of the hybrid immunity [16]. 

Moreover, the anti-S1 antibody levels maintained increased in the PI group after 6 months of vaccination (data not-shown). Another work studying the immune response after a single dose of BNT162b2 in elderly subjects showed a humoral response in 93% at 5–6 weeks of vaccination [17]. All the subjects in our NHR cohort were vaccinated following the FDA and EMA recommendations, and after two-doses a total of 96.16% NHR had humoral response.

However, not all the antibodies are equal. The neutralizing antibodies have been described as involved in minimizing virus infection [18]. It has been previously demonstrated humoral response after vaccination with BNT162b2 [19]. A limitation of the present study is the lack of neutralization assay. Nevertheless, an indirect approach suggests an association between antibody levels and capability to neutralize [20,21], where a value of 4160 AU was identified as a neutralizing capability.

In the work of Blain et al [11], no assessment of post vaccination risk infection was performed. Here, the subjects with antibody levels at 2 months of vaccination lower than 715 AU/mL showed a higher risk of positive PCR after vaccination. This level of anti-S1 antibody would correspond to the low-responder group. Nevertheless, the measurement of humoral immunity alone could be limited since a specific T cell response has to be elicited before humoral immunity.

In our cohort, only 17 subjects were positive PCR after full vaccination, with a total of 1.06% hospitalizations. These results confirm the reduction of infections, hospitalizations and deaths described in full-vaccinated subjects [22].

The study of specific T cell response could identify patients who developed immunity against the vaccine despite the lack of serologic level of IgG antibodies [6,7,8,9,10]. The assessment of both humoral and cellular immune response after vaccination has allowed establishing groups of patients with an impaired immune system regarding the ability to respond to the vaccine. Therefore, no responders could be at risk of infection. Moreover, in the elderly, the immune response to vaccination is heterogeneous [10] and, given that the NHR population is elderly for the most part, it makes this group of particular interest to study the immune response globally. This approach (study of cellular and humoral response to the vaccine in NHR) is the one that has been taken into account in the present study to identify non-responding NHRs. We considered that those with anti-S1 IgG antibodies already had specific T cell immunity necessary to produce IgG switching in the germinal center, and only those seronegative for anti-S1 IgG were further studied for specific CD4+ T cells response. Here we describe how the NHR without previous contact before vaccination with total immunity reaches 97.42%. In this study, we cannot confirm the utility of the specific cellular assessment as a tool for identifying NHR at risk of infection after vaccination since there were no significant differences in COVID-19 incidence between cellular responders and non-responders. Another limitation of the study was the lack of a cellular test in those subjects with less than 715 AU/mL. This cutoff value was set after statistical analysis but testing of cellular function was not performed during the study since we only considered the value established by routine laboratory at 50 AU/mL.

## 5. Conclusions

After 6 months of full vaccination, 11.88% of NHR subjects were seronegative, however, 34.0% of them retained a detectable specific cellular immune response. In summary, a total of 94.09% of NHR maintained a detectable immune response after six months of vaccination. Moreover, the specific cellular immune assessment could potentially be used as a second-line tool to test the vaccine immune response in those subjects without humoral response.

## Data Availability

The data presented in this study are available on request from the corresponding author. The data are not publicly available due to privacy.

## Supplementary Materials

The following are available online at https://www.mdpi.com/article/10.3390/vaccines9121429/s1, Figure S1: SARS-CoV-2 specific T cell assessment, Table S1: Demographic and clinical records of the non-S1 antibodies (seronegative) subjects at 2 months after full vaccination. Table S2: Demographic and clinical records of the non-S1 antibodies subjects after 6 months after full vaccination.
