# Supplementary material for: COVID-19 mRNA Based Vaccine Immune-Response Assessment in Nursing Home Residents for Public Health Decision
Source: Vaccines (Basel). 2021 Dec 2;9(12):1429. doi: 10.3390/vaccines9121429 (PMC8703754; doi:10.3390/vaccines9121429)
Supplement: Supplementary file 1 [file vaccines-09-01429-s001.zip › Supplementary Table S1.pdf]

Supplementary Table S1. Demographic and clinical records of the non-S1 antibodies subjects after full vaccination.

	**Humoral Non-responders (n=12)**	**Complete Non-responders (n=12)**	**p-value**
Age (y.o) Mean (SD)	85.8 (6.6)	85.4 (6.0)	NS
Female n (%)	9 (75)	7 (58.3)	NS
**Comorbidities n (%)**			
Hypertension	4 (33.3)	6 (50)	NS
Diabetes	2 (16.7)	3 (25)	NS
Alzheimer	2 (16.7)	3 (25)	NS
Cardiovascular disease	3 (25)	0	NS
Transplant recipient	0	1 (8.3)	NS
Malignancy	2 (16.7)	2 (16.7)	NS
Autoimmune disorder	2 (16.7)	1 (8.3)	NS
Immunosuppressant treatment	1 (8.3)	3 (25)	NS
