# Supplementary material for: COVID-19 mRNA Based Vaccine Immune-Response Assessment in Nursing Home Residents for Public Health Decision
Source: Vaccines (Basel). 2021 Dec 2;9(12):1429. doi: 10.3390/vaccines9121429 (PMC8703754; doi:10.3390/vaccines9121429)
Supplement: Supplementary file 1 [file vaccines-09-01429-s001.zip › Supplementary Table S2.pdf]

**Supplementary Table S2. Demographic and clinical records of the non-S1 antibodies subjects after 6 months after full vaccination.**

| | Humoral Non-responders (n=16) | Complete Non-responders (n=34) | p-value |
|---|---|---|---|
| Age (y.o) Mean (SD) | 84.00 (12.43) | 86.09 (5.63) | NS |
| Female n (%) | 11 (68.75) | 21 (61.76) | NS |
| **Comorbidities n (%)** | | | |
| Hypertension | 8 (50) | 15 (44.18) | NS |
| Diabetes | 8 (50) | 7 (20.59) | 0.034 |
| Alzheimer | 1 (6.25) | 2 (5.88) | NS |
| Cardiovascular disease | 3 (18.75) | 6 (17.65) | NS |
| Transplant recipient | 0 | 0 | NA |
| Malignancy | 1 (6.25) | 2 (5.88) | NS |
| Autoimmune disorder | 2 (12.5) | 3 (8.82) | NS |
| Immunosuppressant treatment | 2 (12.5) | 1 (2.94) | NS |
