# Supplementary figures and images for: COVID-19 mRNA Based Vaccine Immune-Response Assessment in Nursing Home Residents for Public Health Decision
Source: Vaccines (Basel). 2021 Dec 2;9(12):1429. doi: 10.3390/vaccines9121429 (PMC8703754; doi:10.3390/vaccines9121429)

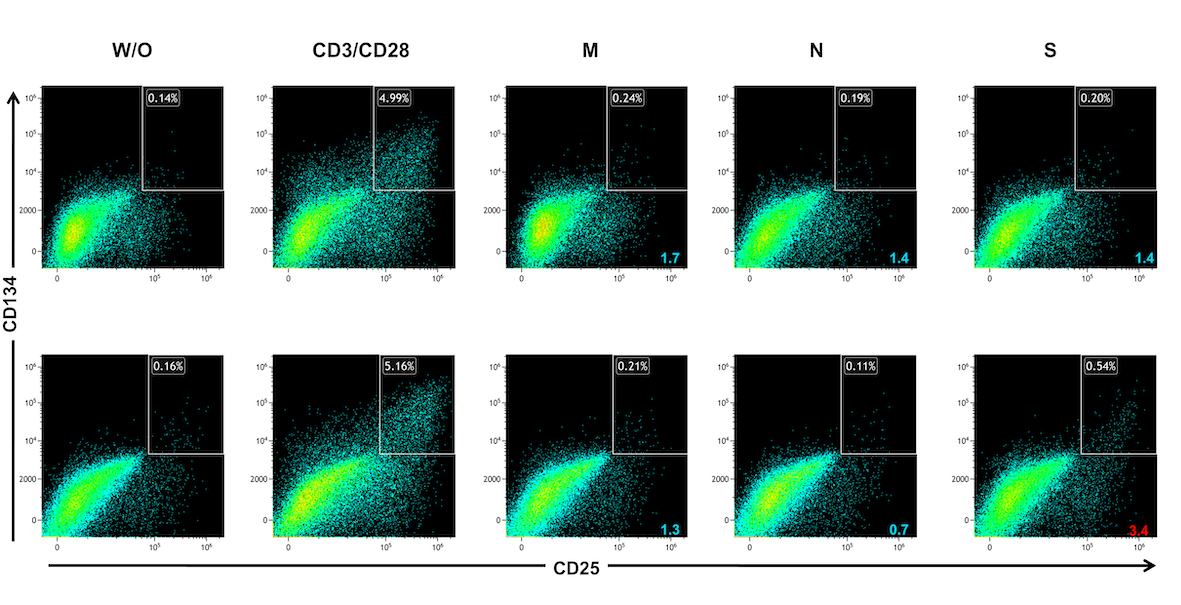

Supplement: Supplementary file 1 [file vaccines-09-01429-s001.zip › Supplementary Figure S1.tif]
